# Superficial Fungal Infections in the Pediatric Dermatological Population of Northern Poland

**DOI:** 10.3390/jof11070533

**Published:** 2025-07-17

**Authors:** Katarzyna Rychlik, Julia Sternicka, Monika Zabłotna, Roman J. Nowicki, Leszek Bieniaszewski, Dorota Purzycka-Bohdan

**Affiliations:** 1 Department of Dermatology, Venereology and Allergology, University Clinical Centre, Medical University of Gdańsk, 80-214 Gdansk, Poland; julia.sternicka@gumed.edu.pl (J.S.); monika.zablotna@gumed.edu.pl (M.Z.); rnowicki@gumed.edu.pl (R.J.N.); 2 Mycology Outpatient Clinic, University Clinical Centre, 80-214 Gdansk, Poland; 3 Clinical Physiology Unit, Medical Simulation Centre, Medical University of Gdańsk, 80-204 Gdansk, Poland; leszek.bieniaszewski@gumed.edu.pl

**Keywords:** superficial fungal infection, dermatophytoses, dermatomycoses, *tinea capitis*, *tinea pedis*, *tinea cutis glabrae*, *onychomycosis*, candidiasis, pediatric, children

## Abstract

Superficial fungal infections (SFIs) remain a common dermatological issue in the pediatric population, with varying prevalence across regions and age groups. This study aimed to assess the epidemiology of SFIs among children and adolescents in northern Poland in the years 2019 to 2024. A retrospective analysis was conducted on 1237 patients under 18 years of age who underwent direct mycological examination and culture, due to suspicion of SFIs. Data were evaluated based on age, gender, infection site, fungal species identified, and place of residence. The prevalence of SFIs in the studied population was 21.4%. The most frequently isolated fungi were *Microsporum canis* and *Trichophyton rubrum complex*. Infection patterns varied by age: *tinea capitis* and *tinea cutis glabrae* predominated in younger children, while adolescents were more affected by *tinea pedis* and *onychomycosis*. A higher proportion of positive results was observed in rural patients, although more urban dwellers were tested. Species distribution also varied with gender and place of residence. No significant change in SFI prevalence or pathogen profile was observed over the study period. This study provides updated insights into the epidemiology of SFIs in Polish children, highlighting the influence of demographic and environmental factors. The findings underscore the importance of accurate diagnosis and suggest a need for further research into behavioral and socio-economic contributors to infection patterns.

## 1. Introduction

Superficial fungal infections (SFIs) are a common clinical issue, diagnosed in patients worldwide [1,2,3]. It is estimated that up to 1/5 of the world’s population may be affected by SFIs [2,4,5]. In the majority, they are caused by dermatophytes and yeasts [2,3,6]. Other infections are rare but can be caused by, for example, molds [2,3].

To make a diagnosis of SFI, clinicians rely largely on the characteristic clinical picture, supplementing the physical examination with mycological tests [5,7,8]. The basic methods of detecting fungal infection are polymerase chain reaction (PCR), direct microscopic examination (DME), and culture on Sabouraud medium with possible additives [5,7,8]. Sometimes, an examination under the light of a Wood’s lamp is also performed [7,8]. Thanks to UV light, it is possible to visualize some species of *Microsporum* (*M.*), for example, *M. audouini* or *M. canis*, which show a green color in fluorescence light [9]. In rare cases, a sample may also be taken and a histopathological examination performed, but this is not the first-choice method [8].

Dermatophytes are the predominant fungi that cause epidermal infections [2,3,6]. The most prevalent representatives of them are the *Trichophyton* (*T.*), *Microsporum*, and *Epidermophyton* genera [4]. They spread through direct or indirect contact with an infected person, animals, or with soil—they can be anthropophilic, zoophilic, or geophilic [2,4,5]. Thanks to their ability to break down keratin, they can invade keratinized parts of the skin, i.e., the epidermis and appendages, causing dermatophytoses [2,10]. Clinical manifestations of such invasions include, among others, *tinea corporis*, *tinea capitis*, *tinea pedis*, *tinea manuum*, *tinea cruris*, and *onychomycosis* [2,4,11].

The second most common group of SFI is candidiasis, which is an infection caused by yeast-like fungi from the *Candida* (*C.*) genus [2,3,9]. Some amounts of *Candida* fungi are present in healthy people, forming part of the saprophytic flora of the skin and mucous membranes [4,12]. However, sometimes, when a disproportion in the amount of fungi occurs, candidiasis develops [4,12]. This usually happens in the mechanism of endogenous infection [4]. Predisposing factors include, among others, immunodeficiencies, both congenital and acquired, or severe systemic diseases [12]. This can lead to the development of *oral* or *vulvovaginal candidiasis*, *candidal balanoposthitis*, *intertrigo*, and many others [4].

Treatment of SFIs can usually be limited to topical preparations such as imidazoles (clotrimazole, ketoconazole, and miconazole), allylamine (terbinafine and naftifine), or ciclopiroxolamine, although in some cases it can be supplemented with oral treatment [7,8,9]. Selecting appropriate medication depends on the drug properties, the host, site of the infection, and the identification of the infectious agent, because the sensitivity of fungi to drugs varies [5,7,8]. An extremely important element of the procedure is follow-up, allowing for possible modification or extension of treatment, which leads to complete resolution of changes and eradication of pathogenic fungi [5].

SFIs are a common dermatological concern not only among adults, but also in pediatric and adolescent populations [13,14,15,16]. Risk factors for SFI include increased sweating, immunocompromised states, diabetes, close contact sports, use of shared facilities such as locker rooms and swimming pools, or hygiene products, like combs or towels [5,14,17]. The etiological factors of pediatric SFIs are comparable to those occurring in adults: the most common are dermatophytes, followed by yeasts, while other fungi are rare in children [17]. The distribution of genus is also similar to that in people over 18 years of age—the most prevalent are *Trichophyton* and *Microsporum* [17]. However, age does affect the frequency of a given species and clinical pictures of fungal infections [18,19]. Adolescents are more commonly affected by infections like *tinea pedis* and *tinea corporis*, due to increased physical activity and exposure, while infants suffer from candida diaper dermatitis or thrush [18,19]. School-age children, on the other hand, account for the majority of cases of *tinea capitis* [17,19]. While the diagnostic methods used to detect fungal species remain unchanged across age groups, the age and weight of the patient must be taken into account when selecting a medication [17].

The objective of this study was to assess the prevalence of superficial fungal infections (SFIs) among pediatric patients in northern Poland, as well as to analyze the association between fungal species and sociodemographic factors, based on data obtained from tests conducted at the Mycological Laboratory of the University Clinical Centre in Gdansk. To date, no study has been conducted on such a large pediatric population in the Pomeranian region of Poland.

## 2. Materials and Methods

Retrospective analysis of the histories of patients diagnosed in the Mycology Outpatient Clinic of the University Clinical Centre in Gdansk, obtained from the patients’ records, has been conducted. At least one visit to the Mycology Outpatient Clinic of the University Clinical Center in Gdansk in the years 2019–2024, referred by dermatologists with suspicion of superficial fungal infection, and the age of the patient being less than 18 years old, was the inclusion criterion. They were met by 1237 pediatric patients.

Patients were tested in specific locations, depending on the symptoms presented. After clinical evaluation, adequate material was collected. Scrapings from the skin and the free rim of the nails were obtained using a sterile scalpel. Hair samples were obtained using tweezers. One part of the collected material was placed on a glass slide for DME. One drop (approximately 0.05 mL) of the mix of 20% solution of potassium hydroxide (KOH) with dimethyl sulfoxide (DMSO) was added to the material on a glass slide to accelerate the penetration of KOH into keratinocytes. The slide was left for 15 min before the examination, so that the KOH solution could dissolve the keratin, and to visualize the fungal structures. The preparation was then evaluated under a microscope (MB-100 microscope; OPTA-TECH, Warsaw, Poland) at 400× magnification by qualified personnel. The second part of the collected material was transferred to a culture plate with Sabouraud medium with chloramphenicol and gentamicin (Graso Biotech, Krąg 4a, Starogard Gdanski, Poland) or Sabouraud medium with chloramphenicol and actidione (Graso Biotech, Krąg 4a, Starogard Gdanski, Poland). Then, after 3 weeks, the culture results were evaluated macro- and microscopically. Macroscopic evaluation of the culture was performed by assessing the color, shape, elevation, consistency, shape of the edges, and smell. In turn, microscopic analysis of the culture consisted of taking a fragment of it with a sterile needle on a basic slide, adding 1 drop of 0.9% NaCl solution, and performing an analysis of morphological features characteristic of a given fungus species, e.g., whether there are macro- or microconidia, spiral hyphae, or chlamydospores. In this way, fungi species were identified and compared with the results of the microscopic examination.

Each visit of a qualified patient was analyzed in terms of the following information: year of the visit, gender, age, and the place of residence of the patient (urban/rural), location of lesions, result of examination in a light microscope, and result of the culture, found species for both methods. The obtained data were subjected to statistical analysis using Statistica 13.3 software (TIBCO Software Inc., Palo Alto, CA, USA, 2017) and SAS 9.4 Release 3.82 (Enterprise Edition), SAS Institute Inc., Cary, NC, USA. The charts were prepared using Microsoft Excel. Analysis of the qualitative features was made with the χ2 test in the Pearson method (it concerns the analysis of test results over the years, depending on gender, place of residence, and location of lesions, taking into account the division into species). The Cochran–Armitage trend test was used to test the null hypothesis that there are no ordered differences in the proportions of positive/negative test results over successive years of observation. The Cochran–Mantel–Haenszel test is applied to test the null hypothesis that the proportions of the fungi aggregated by species are the same over the years, across ordered exposure categories. In all tests, *p*  <  0.05 was considered a significant level of statistical significance.

## 3. Results

**Figure 1:**
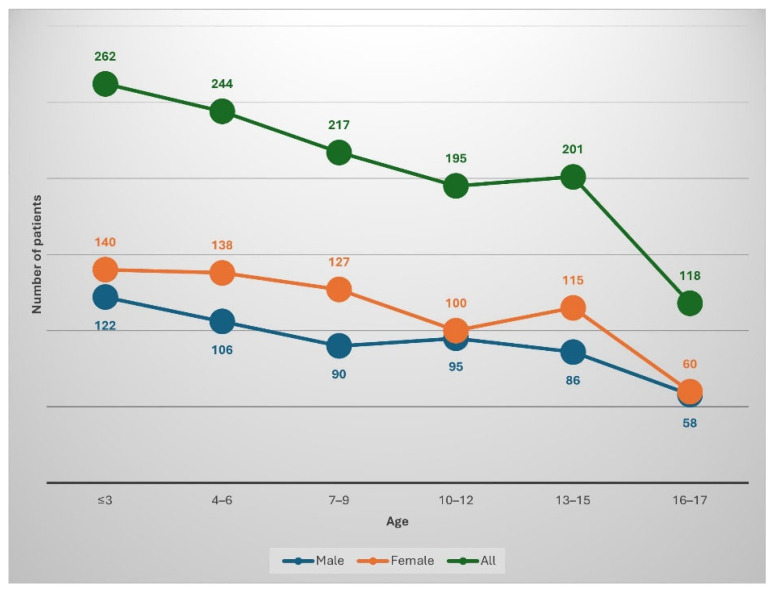
Number of patients in individual age groups by sex, along with the total number of cases in each group.

In the years 2019–2024, 1237 pediatric patients with suspicion of superficial fungal infection were admitted to the Mycology Outpatient Clinic of the University Clinical Centre in Gdansk. Of this group, 557 (45%) were girls, while 680 (55%) were boys. For both girls and boys, the average age of patients was 8.4 years (Figure 1). The majority of the examined patients lived in the city—940 (76%), while a smaller number of them came from the countryside—297 (24%). In the study, 1310 material samples were evaluated, of which in 1297 cases both DME and culture were performed, in 2 cases only culture was performed, and in 11 cases only the DME test was performed. A total of 2607 tests were conducted. The most frequently examined areas were the glabrous skin (42.1%), followed by the scalp (23.5%), toenails (15.5%), fingernails (5.7%), skin of the feet (5%), and skin of the hands (2.8%). The genitals and the skin of the groin were examined just as often (both locations 1.5%), the oral cavity (1.4%), and the area between the toes (1%) were examined least frequently.

**Figure 2:**
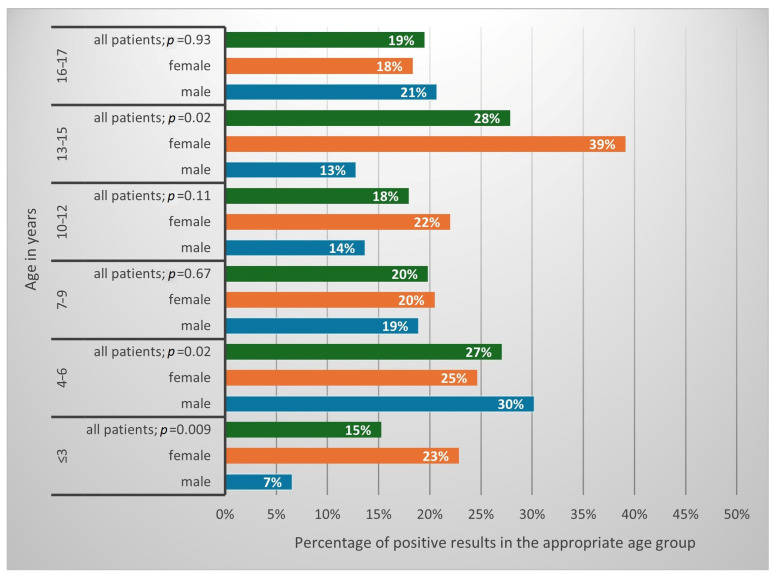
Frequency of positive results in individual age groups, stratified by sex (*p*—Pearson’s chi-squared test).

A positive result of at least one of the tests (DME or culture) was obtained in 265 (21.4%) of patients. This constituted 25.3% of the studied girls and 16.7% of the studied boys (*p* = 0.0003; OR = 1.69, CI 1.27–2.24). The results in percentages, confirming the presence of fungi depending on the patients’ sex and age, are presented in Figure 2.

**Figure 3:**
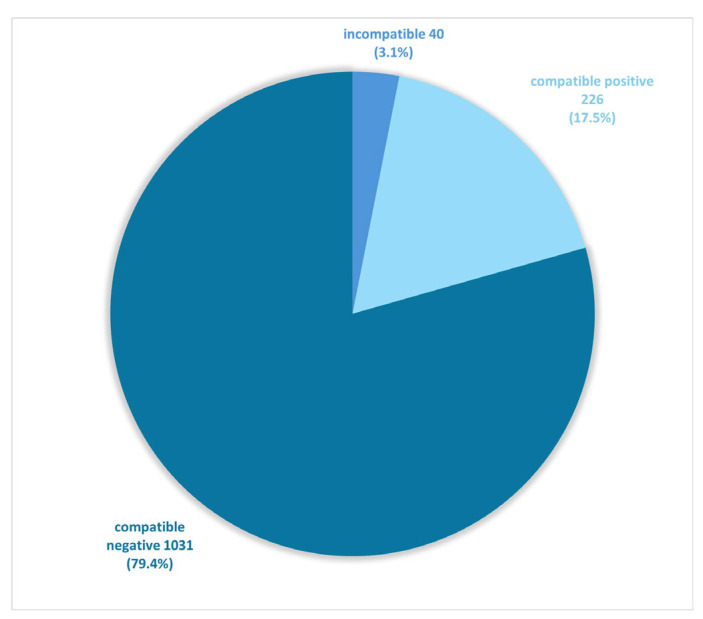
Concordance of DME and culture results (*n* = 1297).

In most cases, DME and culture results were concordant; however, in 3% of studies, these tests showed different results (Figure 3, Table 1 and Table 2). Concerning the incompatible results (*n* = 40), in 22 cases the DME test result was positive and the culture result was negative (55% of incompatible results), while in 18 cases the DME result was negative and the culture result was positive (45% of incompatible results).

The area from which samples taken most often turned out to be positive was the glabrous skin (36.7% of positive tests in different locations, *p* = 0.04), followed by the scalp (35.3%, *p* < 0.0001) and toenails (15.1%, *p* = 0.84). The remaining locations had a smaller share of positive DME and culture results.

**Figure 4:**
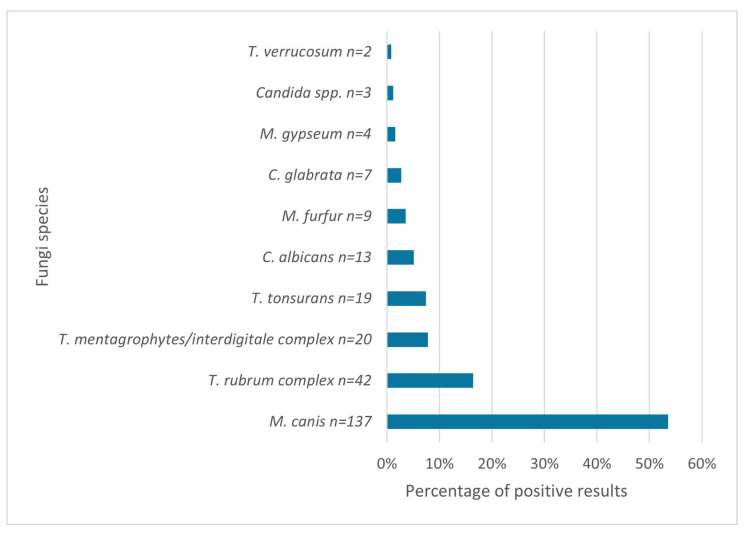
Percentage of particular fungal species in positive test results (*n* = 256; 247 positive cultures and 9 positive DME detecting *Malassezia furfur*).

The most commonly detected group of fungi was dermatophytes (87.5%), followed by yeasts (12.5%); there were no molds found (*p* < 0.0001). The most prevalent species of dermatophytes were *M. canis* and *T. rubrum complex* (Figure 4).

**Figure 5:**
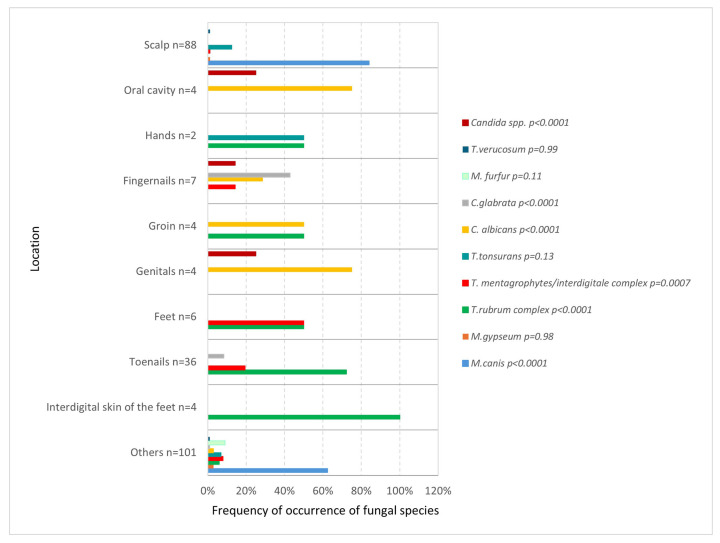
The frequency of occurrence of fungal species by location as determined on the basis of fungi cultures and DMEs (*p* < 0.0001; Pearson’s chi-squared test).

The frequency of occurrence of a particular species varied depending on the test location. On the scalp and body skin, *M. canis* was most common (*p* < 0.0001 and *p* = 0.02), feet were affected equally often by both the *T. rubrum complex* (*p* = 0.02) and the *T. mentagrophytes*/*interdigitale complex* (*p* = 0.0001), while examination of the area of the interdigital spaces and toenails most often revealed the presence of *T. rubrum complex* (*p* < 0.0001, *p* < 0.0001). In the genital area and oral cavity, *C. albicans* predominated (*p* < 0.0001, *p* < 0.0001) (Figure 5).

**Figure 6:**
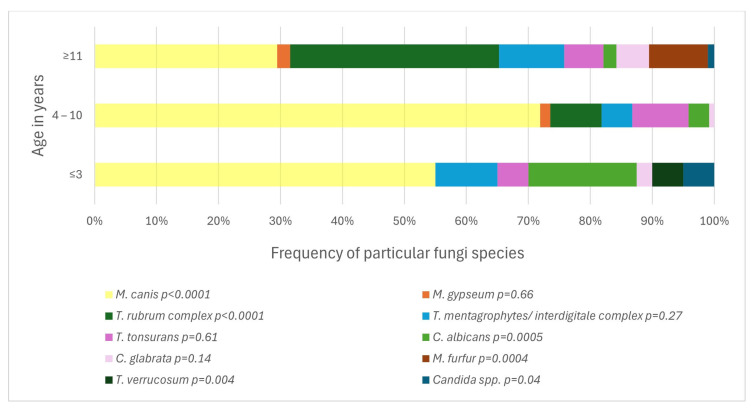
The frequency of particular fungi species in a given age group (*p* < 0.0001; Pearson’s chi-squared test).

The difference between age groups was statistically significant for the frequency of any positive test result (*p* = 0.04) and for the frequency of occurrence of particular fungi species (*p* < 0.0001) (Figure 6). The pathogens associated with age ≤ 3 years of age were *M. canis* (*p* < 0.0001), *T. verrucosum* (*p* = 0.004) and *C. albicans* (*p* = 0.0005). In older children, aged 4–10 years, an association was demonstrated with the presence of *Microsporum canis* (*p* < 0.0001). With age ≥ 11 years, the occurrence of *T. rubrum complex* (*p* < 0.0001) and *Malassezia furfur* (*M. furfur*) (*p* = 0.0004) correlated.

**Figure 7:**
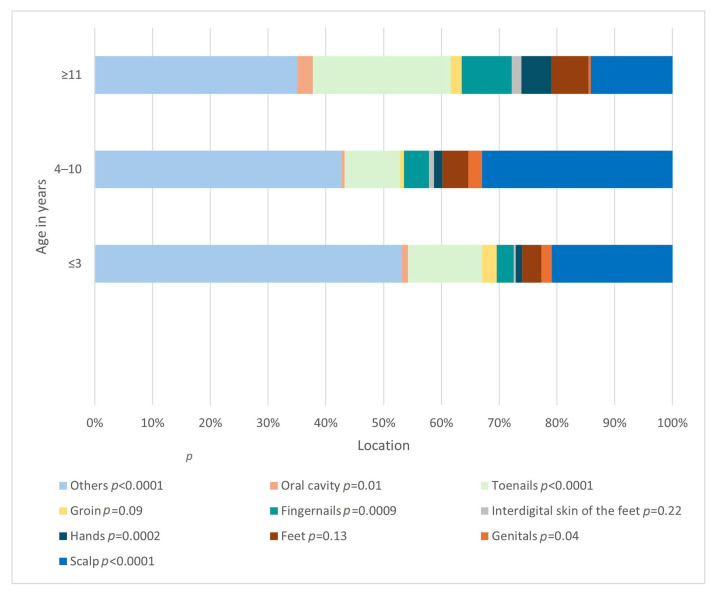
The frequency of involvement of a given location depends on the age group (*p* < 0.0001; Pearson’s chi-squared test).

The difference in the frequency of involvement of a given location also differed significantly (*p* < 0.0001) depending on the age group (Figure 7). The relationships between the age of 4–10 years and the locations: glabrae skin (*p* < 0.0001), scalp (*p* < 0.0001), genital area (*p* = 0.04), as well as the age of >= 11 years and the skin of hands (*p* = 0.0002), fingernails (*p* = 0.0009) and toenails (*p* < 0.0001), were found to be statistically significant.

**Figure 8:**
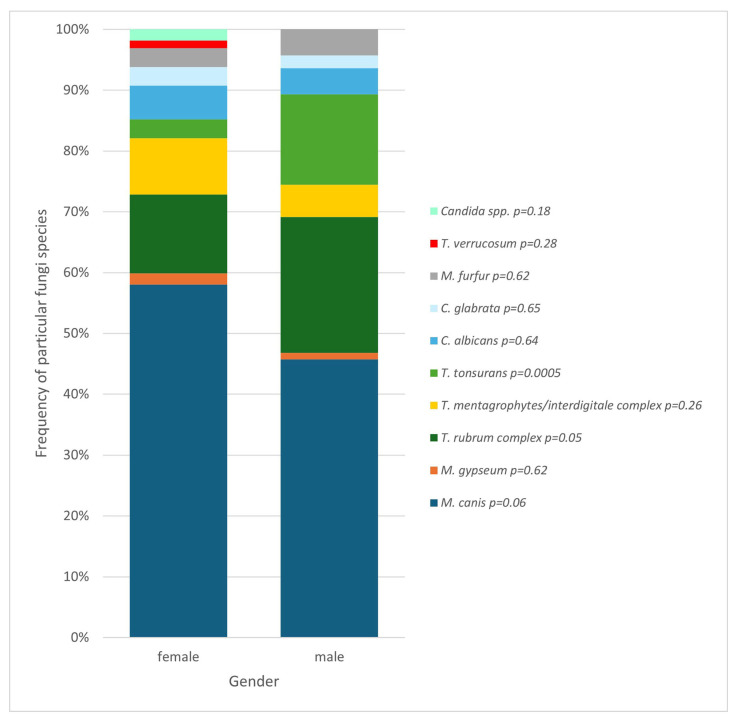
Comparison of fungal species found in girls and boys (*p* = 0.01; Pearson’s chi-squared test).

Relationships have also been found also depend on gender—girls and boys had different frequencies of presence of specific fungi species (Figure 8). The species with a confirmed correlation with gender was proven to be *T. tonsurans*, which correlates with male gender (*p* = 0.0005).

In the context of place of residence, more positive results were obtained by urban dwellers than by rural ones—in patients from urban areas, the presence of SFIs was detected 185 times (72.3%), while for rural residents it was 71 times (27.7%). This represented 18.6% of tests conducted among urban residents and 22.4% of tests conducted among rural residents.

**Figure 9:**
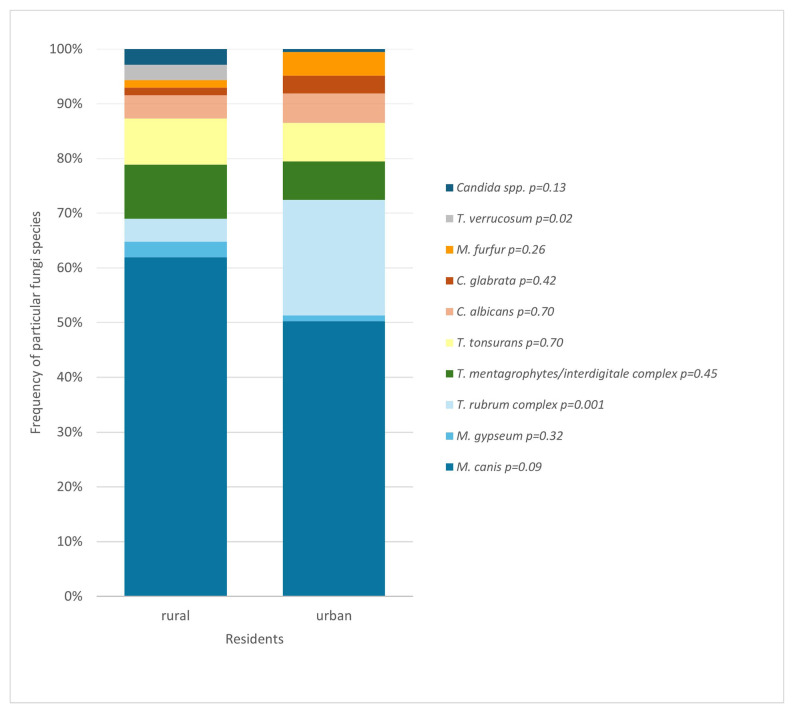
Comparison of fungal species found among rural and urban residents (*p* = 0.01; Pearson’s chi-squared test).

The analysis showed that rural and urban residents differ in the prevalence of occurrence of individual fungi species (Figure 9). The presence of *T. rubrum complex* was found to be associated with living in a city (*p* = 0.001), and *T. verrucosum* with residing in a rural area (*p* = 0.02).

**Figure 10:**
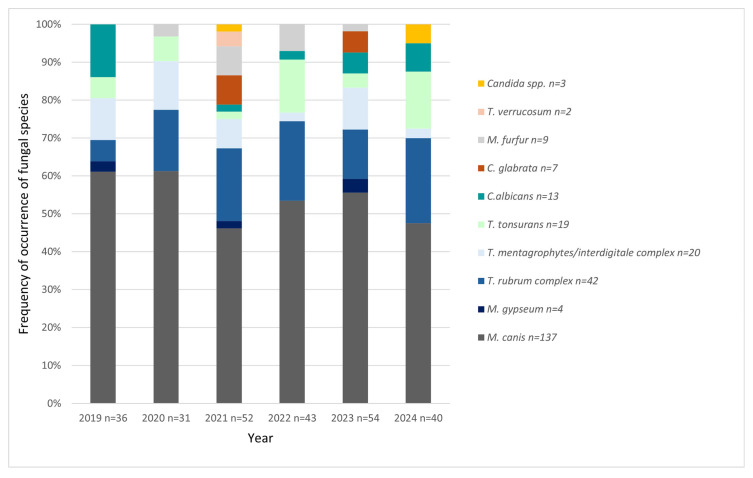
Qualitative distribution and number of positive fungal species detections from 2019 to 2024 (*n*—number of positive results).

The quantitative and qualitative distribution of results over the years was also investigated (Figure 10). The analysis of trend in the data after aggregation to the species (3 levels: *Microsporum* spp., *Trichophyton* spp., and *Candida* spp./other) using Cochran–Mantel–Haenszel test did not show statistical significance (*p* = 0.4410).

The percentage of positive DME and culture results over the years was also analyzed (Table 3). There was no statistically significant trend in the percentage of positive tests, either DME (*p* = 0.913) or culture (*p* = 0.5874), over the years as shown by the Cochran–Armitage test.

## 4. Discussion

The prevalence of SFIs varies, depending on the population studied. This article provides information on SFIs spread in the Northern Polish dermatological population under 18 years of age, estimating the prevalence of these dermatoses at 21.4%. In a research conducted in Nigeria, among 1662 children included in the analysis, the overall frequency of SFIs detection was 45%, with urban dwellers having a higher rate (60.4%) compared to rural ones (29.6%) [20]. An investigation conducted on a smaller group by Gandhi et al. indicated that the prevalence of dermatophytosis in the pediatric population was 19% [21]. A five-year retrospective study carried out on patients of the Mycology Laboratory of Shiraz Medical School showed a low prevalence of SFIs among children, without providing exact numbers [2]. A research examining the epidemiology of SFIs in the Polish population, run by Jaworek et al., also does not provide the percentage of minors affected by these diseases [22].

In 3% of cases, DME and culture results were discordant (Figure 3). This may be due to false negative culture results after the use of drugs such as antifungals or steroids, which suppress immune cell activity allowing fungi to disseminate or persist in low-activity forms, leading to fewer viable organisms at the sampling site, but sometimes also to the lack of fungal hyphae in the material selected for DME testing [2].

It should also be remembered that not every fungus can be identified in culture on classic Sabouraud agar—in the case of *M. furfur*, the result of DME and sometimes examination in the Woods lamp are sufficient [5,7,8]. This emphasizes that the use of both of these methods in each patient is an important element, ensuring diagnostic accuracy. Thanks to this, we increase the patient’s chances for a correct diagnosis and appropriate treatment.

Dermatophytes were the most frequently identified fungi, followed by yeasts, while no molds were detected, which is consistent with publications from many different countries [2,3,4,22]. The most frequently detected species was *M. canis*, which is concordant with another study conducted in northern Poland [23]. The dominance of this fungus has been maintained in the Pomerania region for many years [24], but also in other European countries [19], although many publications indicate other species of fungi as the most common [4]. The second most prevalent species was *T. rubrum complex*, which is also consistent with previous reports from this area [23]. An interesting observation is the appearance of *T. tonsurans*, which had not been observed in the study area in the years 1999–2001 [23]. This is probably due to increased human migration and thus the transfer of pathogens from other areas.

The distribution of fungal species varied according to the anatomical site of sampling. *M. canis* was the predominant species isolated from the scalp and body skin, whereas *T. rubrum complex* was most frequently detected in samples obtained from interdigital spaces of the feet and toenails. In contrast, *C. albicans* was the most commonly identified species in the genital region and oral cavity. Other publications indicate a similar relationship—*M. canis* is often indicated as the main causative agent of *tinea capitis* [4,19,22,23,25,26], and *T. rubrum complex* as the main pathogen causing *tinea pedis* [4,17,22,26]. Although there are some differences—some studies confirm *M. canis* as the leading pathogen causing *tinea cutis glabrae* [19,27], but others point to *T. rubrum complex* as the main fungus [4,17,26]. Moreover, *C. albicans* is usually indicated as more prevalent in fingernail infections [28].

By analyzing the available information from a different perspective, one can also find important data on the location of SFIs. The most common areas occupied by pathogenic fungi in the studied population were glabrous skin, scalp, and toenails, i.e., *tinea cutis glabrae*, *tinea capitis*, and *tinea unguium* (*onychomycosis*), respectively. This is standard in the European population [4,26] and is coherent with previous reports from Gdansk, with one exception—the share of *tinea pedis* decreased significantly [4,23].

Analysis also showed a different prevalence of SFIs in the pediatric population depending on sex, with a positive mycological test result found in over 23% of females and less than 14% of males. Some studies have proven an inverse correlation, i.e., a higher prevalence among boys [20,29,30,31], but a large part of the publications, including one conducted in Poland, showed results consistent with those reported in this report [2,32,33,34,35]. The observed difference could be due to several factors. It may be caused by sociodemographic aspects of the study group—a research conducted by Ezomike et al. showed that urban girls were more likely than boys to be affected by SFIs [20]. In this study, and one conducted in Lodz, Poland [33], the majority of the group were people living in a town, so the discussed finding may be the result of sociodemographic factors. Another component is the fact that girls and boys may have different gender roles and behaviors that affect their likelihood of being exposed to fungal infections, depending on the part of the world. For example, girls in Western culture might be more likely to wear tights or tight footwear, which could contribute to a higher rate of infections like *tinea pedis* or candidiasis. In other regions, for example, in Nigeria, men are more likely to work outdoors and have more contact with the soil and animals, which may act as a reservoir for pathogenic fungal species and constitute a source of infection [20]. However, this work fails to provide a thorough analysis of the factors causing the dissimilarity in SFIs’ prevalence depending on gender in different cultures, so further research is needed to precisely determine the cause of this inconsistency. Another discovery from this research is the statistically significant variation in species distribution based on gender. *T. tonsurans* was detected more often in boys, showing a significant correlation of this species with the male gender. Tests also revealed more often the presence of *T. rubrum complex* among boys and *M. canis* among girls; nevertheless, those were not statistically significant observations (Figure 8). However, a publication in *The Journal of Dermatology* confirms these relationships [19].

An issue of no lesser influence, both on the prevalence of fungal infections and their etiological factor, is the age of the patients. The likelihood of fungal infection correlates with age, which is concordant with different studies on that matter (Figure 2) [15,19,23]. Also, the type of most common SFIs pathogens changes with age (Figure 6). In children ≤ 3 years old, the most common is *M. canis*, but *C. albicans* and *T. verrucosum* also show a correlation with this age group. In older children, *M. canis* predominates. In turn, among teenagers, *T. rubrum complex* and *M. furfur* are statistically more prevalent. This is consistent with the publications of Zienicke et al. and Lange et al., who also proved an analogous relationship for *M. canis* and *T. rubrum complex* [19,23]. However, they did not show that *T. verrucosum* was common in children ≤ 3 years of age, and they did not address the issue of the prevalence of *Candida* by age. This indicates that the contribution of individual species to causing SFIs in specific age groups in children changes over time, and therefore, new research provides valuable insight into the current epidemiological situation in the study area.

The highest percentage of positive results among the tests performed was found in the 13–15 and 4–6 age groups, while the lowest was in the ≤3 age group, which correlates with previous studies on SFIs in the pediatric population in Gdansk [23]. Therefore, it can be seen that the accuracy of the clinical suspicions of the doctors referring the patient for the test varies depending on age, but no statistical correlation is present.

Another aspect that changes depending on the age of the patient is the location of the infection, which is another fact confirmed by other publications [26,36]. The majority of cases involving the glabrous skin, scalp, and genitals were observed in individuals aged 4 to 10 years of age. In contrast, the largest proportion of involvement of the skin of the hands and fingernails, as well as toenails, was observed in the group from 11 years of age. Looking at it from another perspective, in each age group, SFIs were most often located on glabrous skin. Kovitwanichkanont confirmed that *tinea capitis* was most commonly encountered in prepubertal children [36]. Age distribution of *tinea cutis glabrae* also aligns with previous observations, which indicate that it tends to be particularly prevalent in late childhood and early adolescence [23]. The occurrence of fingernail and toenail mycosis in adolescents, rather than younger children, in whom the occurrence of these SFIs is sporadic, is consistent with the literature as well [26]. According to Hawkins et al., *tinea pedis* is typically seen in teenagers, which is in line with the age-related distribution observed in our study [17].

An important factor influencing the frequency of SFIs detection in patients is their place of residence. In this study, although the absolute number of positive tests was higher among urban dwellers, a higher percentage of rural patients received results confirming the presence of fungi. It was proven that the prevalence of SFIs shows a statistically significant correlation with place of residence (*p* = 0.009). This may be due to many factors, such as hygiene practices, access to sanitary facilities, different professional duties, and therefore dissimilarities in clothing, as well as greater exposure to soil or contact with animals [37,38,39,40]. Unfortunately, the above thesis is based on other publications [37,38,39,40], because one of the limitations of this work is the lack of data on such environmental factors as living conditions, footwear, contact with soil or animals, and family history of fungal infections. Another important conclusion can be drawn from these results—town dwellers, despite lower exposure to environmental factors conducive to the development of SFIs, were tested more often. This may be related to the location of the mycological laboratory, the test results of which were analyzed in this work—it is located in a city. However, it may also indirectly indicate another problem—inequality in access to health services for people from rural areas.

A different observation that can be inferred from the results of this study is the variability of species depending on the place of residence (Figure 9). The species associated with urban areas turned out to be *T. rubrum complex*. In turn, living in the countryside correlated with an increased frequency of *T. verrucosum*. In both of those cases, the link is statistically significant.

Within the scope of this study, there is a lack of significant trends in the number of positive tests over the years (Figure 10). The analysis indicates that not only does the number of SFIs remain constant, but also the pathogens that cause them do not change significantly over time. This lack of either decline or growth over the years 2019–2024 and species stability of fungi causing SFIs suggests constant infection rates and effectiveness of public health measures. These results are interesting in the context of work conducted within the same Clinic in the years 1999–2001 [23]. At that time, a total of 94 cases were found, which averages about 31 cases per year [23]. As can be seen in Figure 10, in this study, there were only two years when the number of cases of fungal infections dropped below 40. This suggests that although our publication does not show this, over a period of years greater than that covered by this study—from 2000 to 2025—there may have been an increase in the number of diagnoses of SFIs in children. This may be due to, among other things, the improvement in the quality of healthcare in Poland, as well as the increased flow of patients through the Clinic in question over the years, so one should refrain from stating that the prevalence of SFIs has also increased. However, it is an interesting observation that could be subjected to in-depth analysis in future studies.

This study has some limitations that should be considered when interpreting the findings. First, due to its retrospective design and reliance on paper-based medical records, there is a possibility of incomplete or inconsistent data, which may have influenced the accuracy of certain clinical or demographic variables. Additionally, the identification of dermatophyte species was based exclusively on morphological and culture-based methods, without the use of molecular techniques such as HIF-1α gene analysis or multilocus sequencing. As a result, precise species-level classification was not feasible, particularly in distinguishing between closely related organisms. For this reason, we have referred to the *T. rubrum complex* and the *T. mentagrophytes*/*interdigitale complex* throughout the manuscript. This limitation affects the specificity of the etiological conclusions and highlights the need for molecular confirmation in future studies.

## 5. Conclusions

Superficial fungal infections (SFIs) in children and adolescents, though typically non-severe, continue to pose a relevant public health concern. This study confirmed that the prevalence and distribution of SFIs are influenced by factors such as age, gender, and place of residence. Notably, rural patients had a higher proportion of positive results despite more frequent testing in urban settings, suggesting potential disparities in healthcare access. The findings also indicate a consistent pattern in both the number of cases and the dominant fungal species over recent years, with *M. canis* and *T. rubrum complex* remaining the most prevalent pathogens. Age-specific and site-specific variation in fungal species was observed, and some gender-related differences in pathogen distribution were noted. These insights highlight the importance of maintaining accurate diagnostic practices and call for further research into environmental, behavioral, and socio-demographic factors shaping the epidemiology of SFIs. Continued focus on early detection, targeted antifungal treatment, and public health interventions will be key to optimizing management and reducing the burden of these infections in the pediatric population.

## Figures and Tables

**Table 1:** The distribution of direct microscopic examination (DME) results depending on the patients’ age and sex.

Sex	DME Result	≤3	4–6	7–9	10–12	13–15	16–17	Total
Female	Total	145	140	134	105	125	63	712
	Positive	31	30	26	19	46	10	162 (22.75% of females)
Male	Total	127	114	95	105	92	63	596
	Positive	8	30	18	14	11	14	95 (15.94% of males)
All	Total	272	254	229	210	217	126	1308 *
	Positive	39	60	44	33	57	24	257 (19.65% of total)

* 1308 tests (1297 tests—both DME and culture, and 11 tests—only DME).

**Table 2:** The distribution of culture results depends on the patients’ age and sex.

Sex	Culture Result	≤3	4–6	7–9	10–12	13–15	16–17	Total
Female	Total	146	140	134	106	120	63	709
	Positive	32	31	26	20	36	12	157 (22.14% of females)
Male	Total	127	114	94	104	90	61	590
	Positive	8	33	16	13	10	10	90 (15.25% of males)
All	Total	273	254	228	210	210	124	1299 *
	Positive	40	64	42	33	46	22	247 (19.01% of total)

* 1299 tests (1297 tests—both DME and culture, and 2 tests—only culture).

**Table 3:** The percentage of positive DME and culture results over the years 2019–2024.

Years	All DME Results	Positive DME Results	Percentage of Positive Results Among All DME Tests	All Cultures	Positive Cultures	Percentage of Positive Results Among All Culture Tests
2019	203	40	19.70%	204	36	17.65%
2020	191	31	16.23%	189	30	15.87%
2021	218	49	22.48%	214	48	22.43%
2022	212	42	19.81%	209	40	19.14%
2023	268	51	19.03%	267	53	19.85%
2024	216	44	20.37%	216	40	18.52%

## Data Availability

The original contributions presented in this study are included in the article. Further inquiries can be directed to the corresponding authors.

## Abbreviations

The following abbreviations are used in this manuscript:

DME	Direct Microscopic Examination
DMSO	Dimethyl Sulfoxide
KOH	Potassium Hydroxide
PCR	Polymerase Chain Reaction
SFIs	Superficial Fungal Infections

## References

[1] Aissat F.Z., Denning D.W. (2023). Fungal infections in Algeria. Mycoses.

[2] Khodadadi H., Zomorodian K., Nouraei H., Zareshahrabadi Z., Barzegar S., Zare M.R., Pakshir K. (2021). Prevalence of superficial-cutaneous fungal infections in Shiraz, Iran: A five-year retrospective study (2015–2019). J. Clin. Lab. Anal..

[3] Cai W., Lu C., Li X., Zhang J., Zhan P., Xi L., Sun J., Yu X. (2016). Epidemiology of Superficial Fungal Infections in Guangdong, Southern China: A Retrospective Study from 2004 to 2014. Mycopathologia.

[4] Havlickova B., Czaika V.A., Friedrich M. (2008). Epidemiological trends in skin mycoses worldwide. Mycoses.

[5] Begum J., Mir N.A., Lingaraju M.C., Buyamayum B., Dev K. (2020). Recent advances in the diagnosis of dermatophytosis. J. Basic Microbiol..

[6] Ameen M. (2010). Epidemiology of superficial fungal infections. Clin. Dermatol..

[7] Rezabek G.H., Friedman A.D. (1992). Superficial fungal infections of the skin. Diagnosis and current treatment recommendations. Drugs.

[8] Howell S.A. (2023). Dermatopathology and the Diagnosis of Fungal Infections. Br. J. Biomed. Sci..

[9] Al Sogair S., Hay R.J. (2000). Fungal infection in children: Tinea capitis. Clin. Dermatol..

[10] Deng R., Wang X., Li R. (2023). Dermatophyte infection: From fungal pathogenicity to host immune responses. Front. Immunol..

[11] Naseri A., Fata A., Najafzadeh M.J., Shokri H. (2013). Surveillance of dermatophytosis in northeast of Iran (Mashhad) and review of published studies. Mycopathologia.

[12] Mayer F.L., Wilson D., Hube B. (2013). Candida albicans pathogenicity mechanisms. Virulence.

[13] Rayala B.Z., Morrell D.S. (2017). Common Skin Conditions in Children: Skin Infections. FP Essent..

[14] Cosio T., Valsecchi I., Gaziano R., Campione E., Botterel F. (2025). Glycation of Nail Proteins as a Risk Factor for Onychomycosis. Comment on Gupta et al. Diabetic Foot and Fungal Infections: Etiology and Management from a Dermatologic Perspective. *J. Fungi* 2024, *10*, 577. J. Fungi..

[15] Fulgence K.K., Marie K.P.C., Akoua V.B., Massafoma K.E.G., Etienne A.K., Abibatou K., Henriette V.A., Sebastien M.A.J., Vincent D., William Y. (2023). Dermatophytosis and the associated risk factors among primary school children in southern and central Côte d’Ivoire. Mycoses.

[16] Rudy S.J. (1999). Superficial fungal infections in children and adolescents. Nurse Pract. Forum..

[17] Hawkins D.M., Smidt A.C. (2014). Superficial fungal infections in children. Pediatr. Clin. N. Am..

[18] Gupta A.K., MacLeod M.A., Foley K.A., Gupta G., Friedlander S.F. (2017). Fungal Skin Infections. Pediatr. Rev..

[19] Zienicke H.C., Korting H.C., Lukacs A., Braun-Falco O. (1991). Dermatophytosis in children and adolescents: Epidemiological, clinical, and microbiological aspects changing with age. J. Dermatol..

[20] Ezomike N.E., Ikefuna A.N., Onyekonwu C.L., Ubesie A.C., Ojinmah U.R., Ibe B.C. (2021). Epidemiology and pattern of superficial fungal infections among primary school children in Enugu, south-east Nigeria. Malawi Med. J..

[21] Gandhi S., Patil S., Patil S., Badad A. (2019). Clinicoepidemiological Study of Dermatophyte Infections in Pediatric Age Group at a Tertiary Hospital in Karnataka. Indian J. Paediatr. Dermatol..

[22] Jaworek A.K., Hałubiec P., Sroka D., Grabarczyk I., Kachnic N., Wojas-Pelc A., Szepietowski J.C. (2024). Demographic and Pathogen Profiles of Superficial Fungal Infections-A Single-Centre Observational Study in Poland. Mycoses.

[23] Lange M., Nowicki R., Barańska-Rybak W., Bykowska B. (2004). Dermatophytosis in children and adolescents in Gdansk, Poland. Mycoses.

[24] Wilkowska A., Siedlewicz A., Nowicki R., Szarmach A., Szarmach H. (1995). Grzybica skóry u dzieci w latach 1984–1993. Mikol. Lek..

[25] Ginter-Hanselmayer G., Weger W., Ilkit M., Smolle J. (2007). Epidemiology of tinea capitis in Europe: Current state and changing patterns. Mycoses.

[26] Gawdzik A., Nowogrodzka K., Hryncewicz-Gwóźdź A., Szepietowski J., Maj J., Jankowska-Konsur A. (2021). Epidemiology of dermatophytoses in paediatric population in Southwestern Poland, 2011–2016. Postep. Dermatol. Alergol..

[27] Jeske J., Lupa S., Seneczko F., Głowacka A., Ochęcka-Szymańska A. (1999). Epidemiology of Dermatomycoses of Humans in Central Poland. Part V. Tinea Corporis. Mycoses.

[28] Gawdzik A., Nowogrodzka K., Hryncewicz-Gwóźdź A., Maj J., Szepietowski J., Jankowska-Konsur A. (2019). Epidemiology of dermatomycoses in southwest Poland, years 2011–2016. Postep. Dermatol. Alergol..

[29] Yoon H.J., Choi H.Y., Kim Y.K., Song Y.J., Ki M. (2014). Prevalence of fungal infections using National Health Insurance data from 2009-2013, South Korea. Epidemiol. Health.

[30] Gamage H., Sivanesan P., Hipler U.C., Elsner P., Wiegand C. (2020). Superficial fungal infections in the department of dermatology, University Hospital Jena: A 7-year retrospective study on 4556 samples from 2007 to 2013. Mycoses.

[31] Berenji F., Mahdavi Sivaki M., Sadabadi F., Andalib Aliabadi Z., Ganjbakhsh M., Salehi M. (2016). A retrospective study of cutaneous fungal infections in patients referred to Imam Reza Hospital of Mashhad, Iran during 2000–2011. Curr. Med. Mycol..

[32] de Albuquerque Maranhão F.C., Oliveira-Júnior J.B., Dos Santos Araújo M.A., Silva D.M.W. (2019). Mycoses in northeastern Brazil: Epidemiology and prevalence of fungal species in 8 years of retrospective analysis in Alagoas. Braz. J. Microbiol..

[33] Lupa S., Seneczko F., Jeske J., Głowacka A., Ochecka-Szymańska A. (1999). Epidemiology of dermatomycoses of humans in central Poland. Part IV. Onychomycosis due to dermatophytes. Mycoses.

[34] Moubasher A.H., Abdel-Sater M.A., Soliman Z. (2017). Incidence and biodiversity of yeasts, dermatophytes and non-dermatophytes in superficial skin infections in Assiut, Egypt. J. Mycol. Med..

[35] Araújo Gde M., Araújo N.D., Farias R.P., Cavalcanti F.C., Lima Mdo L., Braz R.A. (2010). Superficial mycoses in Paraíba: A comparative analysis and bibliographical revision. An. Bras. Dermatol..

[36] Kovitwanichkanont T., Chong A.H. (2019). Superficial fungal infections. Aust. J. Gen. Pract..

[37] Sy O., Diongue K., Ba O., Ahmed C., Elbechir M., Abdallahi M., Brahim M., Baidy B., Ndiaye D. (2021). Tinea capitis in school children from Mauritania: A comparative study between urban and rural areas. J. Mycol. Med..

[38] Keerthi S., Punuru L. (2018). A study on prevalence of superficial fungal infections in children in a tertiary care center. Int. J. Dermatol. Venereol. Lepr. Sci..

[39] Seebacher C., Bouchara J.P., Mignon B. (2008). Updates on the epidemiology of dermatophyte infections. Mycopathologia.

[40] Al-Fouzan A.S., Nanda A., Kubec K. (1993). Dermatophytosis of children in Kuwait: A prospective survey. Int. J. Dermatol..

